# Supplementary material for: Phenotypical and Functional Alteration of γδ T Lymphocytes in COVID-19 Patients: Reversal by Statins
Source: Cells. 2022 Oct 31;11(21):3449. doi: 10.3390/cells11213449 (PMC9656060; doi:10.3390/cells11213449)
Supplement: Supplementary file 1 [file cells-11-03449-s001.zip › cells-1970571-supplementary.pdf]

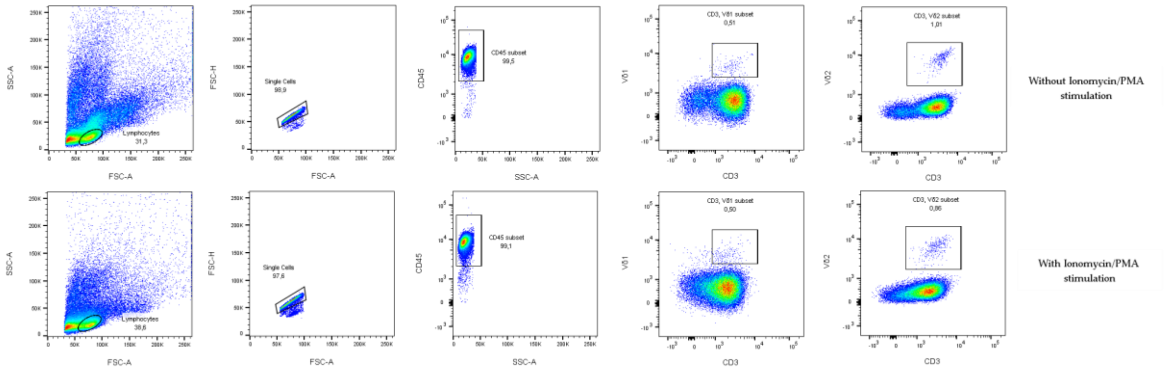

Supplementary Figure S1: Gating strategy for the identification of γδ T cells before and after Ionomycin/PMA stimulation.
